# Child overweight in general practice – parents’ beliefs and expectations – a questionnaire survey study

**DOI:** 10.1186/1471-2296-14-152

**Published:** 2013-10-11

**Authors:** Merethe K Andersen, Bo Christensen, Jens Søndergaard

**Affiliations:** 1 Department of General Practice, Department of Public Health, Aarhus University, Bartholins Allé 2, Aarhus C, Denmark; 2 Institute of Public Health, University of Southern Denmark, J. B. Winsløws Vej 9A, Odense C, Denmark

**Keywords:** Children, Overweight, Parents, Beliefs, Expectations, General practice

## Abstract

**Background:**

Care for overweight children in general practice involves collaboration with parents. Acknowledging the parents’ frames of references is a prerequisite for successful management. We therefore aimed to analyse parental beliefs about the presumed causes and consequences of overweight in children and expectations towards the GP. Moreover, we aimed at comparing the beliefs and expectations of parents of non-overweight children (NOWC) and parents of overweight children (OWC).

**Methods:**

A cross-sectional survey. Data were obtained from a questionnaire exploring parents’ beliefs and expectations regarding overweight in children. The questionnaires were completed by parents following their child’s participation in the five-year preventive child health examination (PCHE).

Parental agreement upon statements concerning beliefs and expectations regarding overweight in children was measured on a Likert scale. Differences in levels of agreement between parents of non-overweight children and parents of overweight children were analysed using Chi-squared test and Fisher’s exact test.

**Results:**

Parents of 879 children completed and returned questionnaires. Around three fourths of the parents agreed that overweight was a health problem. A majority of parents (93%) agreed that the GP should call attention to overweight in children and offer counselling on diet and exercise. Almost half of the parents expected a follow-up programme. Parents of overweight children seemed to agree less upon some of the proposed causes of overweight, e.g. inappropriate diet and lack of exercise. These parents also had stronger beliefs about overweight disappearing by itself as the child grows up.

**Conclusions:**

According to parental beliefs and expectations, general practice should have an important role to play in the management of child overweight. Moreover, our findings suggest that GPs should be aware of the particular beliefs that parents of overweight children may have regarding causes of overweight in their child.

## Background

The preventive child health examinations in Danish general practice have been proposed to be a particularly good opportunity to provide prevention, identification of and care for overweight children [1] the participation rate being 83% at the five-year PCHE [2]. Caring for overweight children in general practice presupposes close collaboration between GPs and parents, and the GPs need to understand parents’ beliefs and expectations to tailor effective interventions against child overweight [3]. A study of Australian GPs’ barriers to managing child overweight showed that only a third of the GPs thought that parents perceived overweight in their child to be a health problem and that a possible negative response from the parents when addressing overweight was a barrier to the GPs discussing overweight in the child [4]. Furthermore, another study revealed that parents of overweight children, believing that their child’s weight is a health problem, are more inclined to make changes for their overweight child than those not believing so [5]. Consequently, GPs’ barriers to managing child overweight may partly be unfounded. A hospital setting study revealed that parents of overweight children expected the health professionals to call attention to overweight, although the child was admitted for an acute disease [6]. Concurrently, a previous study showed that overweight was not addressed in one third of overweight children at the five-year PCHE in Danish general practice, [7] although weight assessment is an integral part of the PCHE programme [8]. In order to establish collaboration between GPs and parents regarding child overweight, a common basis is needed. Therefore, it seems requisite for GPs to possess knowledge about parents’ beliefs of the causes and consequences of overweight in children and their expectations towards the GP. However, the beliefs and expectations of parents participating with their child in a PCHE have not been examined.

Interview studies have shown that some parents of overweight children have particular beliefs about the causes of overweight in their own child and imply that many families believe that the child is obese due to factors not related to eating and/or exercise [9] and that some mothers attribute their child’s obesity to family traits, child being “tall or big-boned” or something that would be “grown out of” [10]. These particular beliefs need to be clarified prior to a meaningful dialogue between GPs and parents. If a child’s parents believe that the child is overweight due to “being big boned”, or that the child is overweight because of an infant formula it had several years ago, it makes no sense to the parents when the GP supervises current diet and exercise. To be able to help patients, we need to meet them where they are and GPs may not be aware of such particular interpretations. If obese children are to be helped in the future, it is essential that the families’ own experience and understanding of the problem are taken into account. If these children are to be helped, it is of little value to focus strictly on food and exercise. Instead, it will be of great importance to integrate the families’ own understanding and experience into the management in primary care.

In this study we aimed to quantify how widespread these particular beliefs are, and if they differ between parents of normal weight children (NOWC) and parents of overweight children (OWC). Moreover, we evaluated parents’ expectations towards the GP regarding overweight in children.

## Methods

### Design

We conducted a cross-sectional survey based on questionnaires completed by parents accompanying their child at the five-year PCHE. The questionnaire targeted the parents’ beliefs about the presumed causes and consequences of overweight in children and their expectations towards the GP. They completed the questionnaire after having participated in the five-year PCHE.

### Setting

The Danish healthcare system is tax-financed. More than 98% of the Danes are registered with a GP and receive free medical care [11]. From birth to the age of five years, all Danish children are offered an annual PCHE by their GP.

The aim of the PCHE is combined prevention and health promotion. Weight and height are measured at almost all five-year PCHEs [2].

### Sampling

The study was performed in the Central Denmark Region (1 250 000 inhabitants). In September 2008 all GPs in the region (N=865) were invited to participate in the study and to hand out a questionnaire to all parents who consecutively attended the five-year PCHE, regardless of their child’s weight. Reminders were not sent to the parents, as the potential parent population was not known in advance by the researchers. Parents were supposed to fill in the questionnaire at home and were provided with prepaid return envelopes. The sampling took place from January 2009 to January 2010. The parents had to be able to speak and read Danish. The five-year PCHE was chosen, because it is the last chance for the GPs to identify overweight and initiate intervention before the health examinations are passed to the municipal health care when children start school. Moreover, the participation rate is relatively high and the prevalence of overweight is higher than at the previous PCHEs [2].

### Questionnaire development

This research area is largely uncharted in the general practice setting, which left us without the possibility of deploying validated questionnaires to answer the questions raised in the present study. We therefore developed the items for the questionnaire primarily based on literature studies and [4,12-17] focus group interviews with GPs and parents of overweight children.

The questionnaire was reviewed and pilot-tested by colleagues, researchers, healthcare professionals and laypersons (viz. parents). The pilot tests led to minor changes of the phrasing of the questions, and no questions or themes were subsequently added or dropped.

The final questionnaire comprised five themes of which two themes have been addressed in this paper: The questions are presented in Tables 1, 2, 3 and 4.

**Table 1:** Parents’ beliefs about overweight in children, distributed on NOWC and OWC

				**Agree**	**Slightly**	**Slightly**	**Disagree**	**Don't**	**Missing**	
					**agree**	**disagree**		**know**		
				**n(%)**	**n(%)**	**n(%)**	**n(%)**	**n(%)**	**n(%)**	**p-value**
***Overweight in children is caused***										
***by hereditary conditions***										
	**Weight status**	**N=772**	**NOWC n(%)**	70(9.1)	454(58.8)	134(17.4)	63(8.2)	43(5.6)	8(1.0)	
		**N=87**	**OWC n(%)**	8(9.2)	54(62.1)	10(11.5)	10(11.5)	2(2.3)	3(3.4)	0.37
***Overweight in children is caused by***										
***inappropriate diet***										
	**Weight status**	**N=772**	**NOWC n(%)**	593(76.8)	169(21.9)	3(0.4)	0(0.0)	1(0.1)	6(0.8)	
		**N=87**	**OWC n(%)**	56(64.4)	28(32.2)	0(0.0)	1(1.2)	0(0.0)	2(2.3)	0.02
***Overweight in children is caused by***										
***too little exercise***										
	**Weight status**	**N=772**	**NOWC n(%)**	519(67.2)	237(30.7)	6(0.8)	1(0.13)	2(0.3)	7(0.9)	
		**N=87**	**OWC n(%)**	45(51.7)	35(40.2)	1(1.2)	4(4.6)	0(0.0)	2(2.3)	0.01
***Overweight in children goes away by***										
***itself, as they grow up***										
	**Weight status**	**N=772**	**NOWC n(%)**	5(0.7)	114(14.8)	291(37.7)	338(43.8)	16(2.1)	8(1.0)	
		**N=87**	**OWC n(%)**	3(3.5)	23(26.4)	29(33.3)	25(28.7)	3(3.5)	4(4.6)	0.001
***Overweight in children is a problem***										
***requiring medical care***										
	**Weight status**	**N=772**	**NOWC n(%)**	142(18.4)	415(53.8)	138(17.9)	20(2.6)	49(6.4)	8(1.0)	
		**N=87**	**OWC n(%)**	12(13.8)	42(48.3)	17(19.5)	1(1.2)	10(11.5)	5(0.6)	0.31
***Overweight in children can be reverted into normal weight***										
	**Weight status**	**N=772**	**NOWC n(%)**	612(79.3)	137(17.8)	2(0.3)	3(0.4)	9(1.2)	9(1.2)	
		**N=87**	**OWC n(%)**	64(73.6)	17(19.5)	2(2.3)	0(0.0)	2(2.3)	2(2.3)	0.10

“What beliefs do you have about overweight in children?”

**Table 2:** Parents’ beliefs about consequences of overweight in children, distributed on NOWC and OWC

				**Agree**	**Slightly**	**Slightly**	**Disagree**	**Don't**	**Missing**	
					**agree**	**disagree**		**know**		
				**n(%)**	**n(%)**	**n(%)**	**n(%)**	**n(%)**	**n(%)**	**p-value**
***Problems keeping up with***										
***other children during play***										
	**Weight status**	**N=772**	**NOWC n(%)**	537(69.6)	192(24.9)	14(1.8)	3(0.4)	20(2.6)	6(0.8)	
		**N=87**	**OWC n(%)**	60(69.0)	20(23.0)	1(1.2)	0(0.0)	4(4.6)	2(2.3)	0.76
***Problems with being***										
***bullied***										
	**Weight status**	**N=772**	**NOWC n(%)**	633(82.0)	120(15.5)	4(0.5)	0(0.0)	5(0.7)	8(1.0)	
		**N=87**	**OWC n(%)**	74(85.1)	9(10.3)	0(0.0)	1(1.2)	1(1.2)	2(2.3)	0.27
***Problems with overweight***										
***later in life***										
	**Weight status**	**N=772**	**NOWC n(%)**	666(86.3)	82(10.6)	3(0.4)	3(0.4)	11(1.4)	7(0.9)	
		**N=87**	**OWC n(%)**	71(81.6)	13(14.9)	0(0.0)	0(0.0)	1(1.2)	2(2.3)	0.66
***Problems with heart disease***										
***later in life***										
	**Weight status**	**N=772**	**NOWC n(%)**	600(77.7)	67(77.01)	4(0.5)	2(0.3)	37(4.8)	5(1.0)	
		**N=87**	**OWC n(%)**	124(16.1)	13(14.9)	0(0.0)	0(0.0)	4(4.6)	3(3.4)	1.0
***Problems with diabetes***										
***later in life***										
	**Weight status**	**N=772**	**NOWC n(%)**	589(76.3)	137(17.8)	2(0.3)	2(0.3)	37(4.8)	5(1.0)	
		**N=87**	**OWC n(%)**	67(77.0)	10(11.5)	0(0.0)	0(0.0)	7(8.1)	3(3.4)	0.38

“What problems do you believe that overweight children can have or get?”

**Table 3:** Parents’ expectations to the GPs’ care for overweight in children, distributed on NOWC and OWC

				**Agree**	**Slightly**	**Slightly**	**Disagree**	**Don't**	**Missing**	
					**agree**	**disagree**		**know**		
				**n(%)**	**n(%)**	**n(%)**	**n(%)**	**n(%)**	**n(%)**	**p-value**
***The GP should raise the issue***										
***if a child is overweight***										
	**Weight status**	**N=772**	**NOWC n(%)**	723(93.7)	35(4.5)	0(0.0)	0(0.0)	2(0.3)	12(1.6)	
		**N=87**	**OWC n(%)**	81(93.1)	5(5.8)	0(0.0)	0(0.0)	1(1.2)	0(0.0)	0.26
***The GP should offer counselling***										
***on diet and exercise***										
	**Weight status**	**N=772**	**NOWC n(%)**	534(69.2)	187(24.2)	28(3.6)	3(0.3)	4(0.5)	17(2.2)	
		**N=87**	**OWC n(%)**	58(67.7)	24(27.6)	2(2.3)	2(2.3)	1(1.2)	0(0.0)	0.12
***The GP should offer a programme***										
***for follow-up and treatment***										
	**Weight status**	**N=772**	**NOWC n(%)**	372(48.2)	284(36.8)	68(8.8)	6(0.8)	30(3.9)	12(1.6)	
		**N=87**	**OWC n(%)**	37(42.5)	42(48.3)	3(3.5)	2(2.3)	3(3.5)	0(0.0)	0.08
***The GP should refer to a dietician***										
	**Weight status**	**N=772**	**NOWC n(%)**	430(55.7)	259(33.6)	36(4.7)	2(0.3)	32(4.2)	13(1.7)	
		**N=87**	**OWC n(%)**	40(46.0)	36(41.4)	3(3.5)	2(2.3)	6(6.9)	0(0.0)	0.04
***The GP should refer to a***										
***paediatrician***										
	**Weight status**	**N=772**	**NOWC n(%)**	96(12.4)	281(36.4)	222(28.8)	49(6.4)	107(13.9)	17(2.2)	
		**N=87**	**OWC n(%)**	7(8.1)	30(34.5)	33(37.9	6(6.9)	10(11.5)	1(1.2)	0.45
***The GP should refer***										
***to a health visitor***										
	**Weight status**	**N=772**	**NOWC n(%)**	195(25.3)	333(43.1)	130(16.8)	27(3.5)	71(9.2)	16(2.1)	
		**N=87**	**OWC n(%)**	14(16.1)	40(46.0)	14(16.1)	14(16.1)	5(5.8)	0(0.0)	0.00
***The GP should refer to a psychologist***										
	**Weight status**	**N=772**	**NOWC n(%)**	33(4.3)	174(22.5)	267(34.6)	138(17.9)	144(18.7)	16(2.1)	
		**N=87**	**OWC n(%)**	4(4.6)	18(20.7)	29(33.3)	25(28.7)	10(11.5)	1(1.2)	0.14
***The GP should refer to a municipal***										
***offer for overweight children***										
	**Weight status**	**N=772**	**NOWC n(%)**	277(35.9)	308(39.9)	67(8.7)	16(2.1)	88(11.4)	16(2.1)	
		**N=87**	**OWC n(%)**	28(32.2)	36(41.4)	12(13.8)	6(6.9)	5(5.8)	0(0.0)	0.03

“What expectations do you have to the GP’s care of overweight children?”

**Table 4:** Parents’ beliefs about how parents’ weight should influence on the GP’s role, distributed on NOWC and OWC

				**Agree**	**Slightly**	**Slightly**	**Disagree**	**Don't**	**Missing**	
					**agree**	**disagree**		**know**		
				**n(%)**	**n(%)**	**n(%)**	**n(%)**	**n(%)**	**n(%)**	**p-value**
***The GP should pay attention***										
***regardless of the parents’ weight***										
	**Weight status**	**N=772**	**NOWC n(%)**	725(93.9)	33(4.3)	1(0.1)	0(0.0)	1(0.1)	12(1.6)	
		**N=87**	**OWC n(%)**	78(89.7)	6(6.9)	1(1.2)	0(0.0)	0(0.0)	2(2.3)	0.14
***GP should be more careful***										
***if the parents are overweight***										
	**Weight status**	**N=772**	**NOWC n(%)**	31(4.0)	75(9.7)	112(14.5)	529(68.5)	11(1.4)	14(1.8)	
		**N=87**	**OWC n(%)**	1(1.2)	7(8.1)	15(17.2)	61(70.1)	1(1.2)	2(2.3)	0.7
***GP ought to pay extra attention***										
***to overweight in children of overweight parents***										
	**Weight status**	**N=772**	**NOWC n(%)**	515(66.7)	177(22.9)	36(4.7)	14(1.8)	16(2.1)	14(1.8)	
		**N=87**	**OWC n(%)**	53(60.9)	26(29.9)	2(2.3)	3(3.5)	2(2.3)	1(1.1)	0.34

“How should the parents’ weight have influence on the GP’s role?”

The GP or the practice nurse did the weight and height measurements. The child’s weight was measured (nearest kilo) and height (nearest centimetre) according to current guidelines regarding the conduction of PCHEs in Danish general practice.

In Denmark, most parents bring a “Child’s book” to all health examinations. The GP fills in height, weight and other measurements and comments relevant for the current PCHE. The parents in our study were asked to enter the measurements from the “Child’s book” into the questionnaire. The parents were supposed to fill in their own weight and height measured at home without any guidance and with no validation of the used scales.

The parents registered the identity of the person filling in the questionnaire (“Mother”, “Father”, “Other”), their child’s height and weight (measurements from the five-year PCHE), and the weight and height of both parents (measured by the parents themselves at home without further introduction or validation of the used scales). The parents were asked about the extent to which they agreed with statements regarding beliefs about the presumed causes and consequences of overweight in children (Tables 1 and 2) and their expectations to the GP’s care (Tables 3 and 4). The level of agreement was ticked on a Likert scale (“Agree”, “Slightly agree”, “Slightly disagree”, “Disagree”, “Don’t know”).

### Statistical analysis

GP characteristics were compared between participating and non-participating GPs by using standard (Pearson’s) Chi-squared test. P-values<0.05 were considered statistically significant (Table 5). Data regarding GP characteristics were obtained from the regional authority, which is the GPs’ employer keeping updated data on all GPs in the Central Denmark Region.

**Table 5:** Characteristics of the source population of GPs in the Central Region of Denmark

	**Participating GPs**		**Non-participating GPs**		**p-value**
	**n**	**(%)**	**n**	**(%)**	
	**165**	**(19)**	**700**	**(81)**	
**Gender**					
Male	66	(40)	451	62	<0.001
Female	99	(60)	230	38	
**Age**					
Mean (range)	50.7	(35–65)	53.3	(34–70)	<0.001
Number of conducted five-year PCHEs per year*					
Mean (range)	21	(4–104)	16	(1–80)	0.99
**Type of practice**					
Single-handed practice	38	(23)	184	(26)	0.2
Partnership practice	127	(77)	493	(71)	0.2
Unknown**	0	(0)	23	(3)	
Number of children on list***					
Mean (range)	938	78-3364	830	19-3364	0.99

*Number of conducted PCHEs was assessed on the basis of single-handed practices.

**Type of practice not registered in data from the General Practice Administrative Authorities in the Central.

Denmark Region.

*** The number of children on the list was assessed on the basis of all types of practices.

We categorised the children’s weight status into “Normal weight”, “Overweight” or “Obese” according to Danish standards for BMI in children, [18] which are consistent with the standards used in Danish GPs’ software systems for calculating children’s weight status. The variables “Overweight” and “Obese” were collapsed into one category “Overweight child”. Parental overweight was defined according to WHO guidelines as a BMI above 25.0 kg/m^2^.

Differences in beliefs and expectations between parents of NOWC and of OWC were analysed using Chi-squared test and Fisher’s exact test.

## Results

A total of 865 GPs were invited to participate; 240 (28%) GPs accepted participation and 165 GPs (19%) ended up recruiting parents and children for the study. The participating GPs were statistically significantly younger than the non-participating GPs (Table 5). There were statistically significantly more female GPs among the participating GPs compared to the non-participating GPs. No statistically significant difference in the number of conducted PCHEs between participating and non-participating GPs or between the numbers of children on their lists was found.

A total of 1196 parent questionnaires were handed out by the GPs in relation to the five-year PCHE. Parents of 879 (73%) children completed and returned questionnaires. Mothers filled in 803 (91.4%) of the questionnaires, fathers 73 (8.3%) of the questionnaires. In 3 of the questionnaires, the respondent was not identified.

Height and weight were entered for 859 (97.7%) children of whom 75 (8.7%) children were overweight and 12 (1.4%) children were obese. Height and weight were entered for 857 (97.5%) and 844 (96.0%) mothers, respectively. The father’s height was entered in 804 (91.5%) questionnaires and his weight in 783 (89.1%). A total of 291 (33.1%) of the mothers and 418 (47.6%) of the fathers were overweight. More overweight children (53.2%) than non-overweight children (32.5%) had an overweight mother (p<0.01). More overweight children (75.3%) than non-overweight children (51.6%) had an overweight father (p<0.01).

A high proportion of all parents agreed that overweight in children is caused by inappropriate diet (74%) and too little exercise (64%). A higher proportion of parents of NOWC (76.8%) than of OWC (64.4%) agreed that overweight in children is caused by inappropriate diet (p=0.02). More parents of NOWC (67.2%) than of OWC (51.2%) agreed that overweight in children is caused by too little exercise (p=0.01). Only few parents (0.9%) thought that overweight is going away by itself as children grow up. A smaller proportion of all parents (16%) agreed that overweight in children requires medical attention (Table 1). A higher percentage of parents of OWC than of NOWC agreed with that statement (p=0.001) (Table 1).

A majority of all parents (76%) agreed that overweight in children can be reverted into normal weight. A high proportion of all parents (77%) agreed that child overweight can cause problems like for instance heart disease and diabetes later in life. There were no statistically significant differences between parents of NOWC and of OWC with regard to this (Table 2).

Most of the parents agreed on expecting the GP to raise the issue in case of an overweight child. Moreover, two thirds of all parents agreed that the GP should offer counselling on diet and exercise. Almost one half of the parents expected the GP to offer a follow-up programme.

Half of the parents (51%) agreed that the GP should refer overweight children to a dietician. Statistically significantly more parents of NOWC than of OWC agreed with that statement (p=0.04). Only a small proportion of all parents (10%) agreed that the GP should refer overweight children to a paediatrician (Table 3).

A majority of all parents (92%) agreed that the GP should call attention to overweight in children regardless of the parents’ weight (Table 4). Moreover, two thirds of all parents agreed that the GP should pay more attention to children of overweight parents and that GPs should not be more careful when addressing overweight in children with overweight parents.

## Discussion

A majority of the parents in our study, regardless of their child’s weight status, agreed with statements saying that childhood overweight is a health risk and that the GP should raise the issue of overweight where relevant. Moreover, most of the parents agreed that they expected the GP to offer counselling on diet and exercise. Almost half of the parents agreed on having expectations of a follow-up programme initiated and managed by the GP.

Parents of OWC seemed to have less strong beliefs about inappropriate diet and too little exercise being causes of overweight in children. They were more inclined to agree with a statement saying that overweight will disappear by itself as children grow.

The finding that most parents agreed with statements saying that childhood overweight carries a risk of overweight, heart diseases and diabetes in adulthood is interesting in the light of a study of GPs’ barriers to managing child overweight [4]. That study showed that only 30% of the GPs surveyed thought that parents saw childhood obesity as a health problem. The relevance of our findings is substantiated by a study by Rhee et al., showing that if parents thought that their child’s weight was a health problem, they would be more inclined to make changes for their overweight child than if they did not think so [5].

We found that nearly all parents agreed or slightly agreed with the statement saying that overweight in children is caused by inappropriate diet and too little exercise. However, parents of NOWC tended to agree more with this statement than those of OWC. This is in line with an anthropological study of obese children conducted by Lindelof [9]. Lindelof found that many families believed that the child was obese due to factors not related to eating and/or exercise. An important conclusion in the study by Lindelof was that a child’s overweight is not a result of the family’s lack of knowledge regarding causes of overweight, and it is therefore not possible to help these families only by informing them about proper diet and exercise. Our study implies that parents of OWC may have stronger beliefs about overweight going away by itself as the child grows up. These findings are in line with an interview study of mothers of obese children who attributed their child’s obesity to family traits, the child being “tall or big-boned” or something that they would “grow out of” [10].

A majority of the parents in our study agreed that their GP should adopt an active approach by raising the issue if a child is overweight. This finding is in line with the finding in a study targeting children admitted to a hospital ward [6]. This is interesting seen in the light of a previous study of GPs’ care of overweight at the five-year PCHE, which found that overweight was only addressed in 60% of the children whom the GPs assessed with a weight-for-stature above normal [7]. More studies have shown that parents have mixed experiences with responses from GPs; from being sympathetic to showing lack of interest [19] and that the care of overweight in children is somewhat inadequate [7,13].

### Strengths and limitations

The strength of the present study is that it provides data on a large group of parents of OWC and NOWC participating in a PCHE. The participation rate was high and the prevalence of overweight amongst parents was comparable to that of the background population. The large material enabled us to detect small differences between parents of OWC and of NOWC, which may give rise to a new hypothesis regarding the beliefs of parents of OWC. Since the reason for the encounter was not overweight, we obtained knowledge also on parents of normal weight children as opposed to other studies of parents’ beliefs about childhood overweight [5,7,10,20,21].

A main limitation of the present study is that it draws on a selected group of GPs who were over-represented by females and younger GPs. It is likely that the participating GPs were more committed, which may have biased parental recruitment, probably entailing more aware parents with higher expectations. The participation in PCHEs is in itself associated with selection bias, as non-participant status is characterised by low socioeconomic status, low level of education and by being a single provider [22]. We may hence not have captured knowledge about beliefs and expectations in the less privileged families and we cannot know how selection due to non-participation may have influenced on expectations towards the GP. The lack of demographic data further impairs analyses of this selection.

The GPs were encouraged to hand out a questionnaire to consecutive parents attending the five-year PCHE regardless of their child’s weight. On average, the participating GPs conducted 21 five-year PCHEs per year. Nevertheless, they only included approximately 7 (6.9) children each. This may in part be explained by the fact that the GPs only received 10 questionnaires to begin with. For more inclusions they should request more questionnaires. However, this does not give the full explanation and the lower prevalence of overweight in the studied population (10.1%) compared to the background population (15.9% and 11.6% for 5-8-year-old boys and girls, respectively) [23] implies that selection due to overweight has taken place. This presumption is substantiated by the fact that overweight was more prevalent in children of parents who did not receive a questionnaire (30%).

Some GPs may have encountered barriers to handing out questionnaires to parents with overweight children. Additionally, we cannot know how many parents declined participation, and whether their children were overweight. This selection is of particular importance, as parents of overweight children in our study tended to have different beliefs about the causes and progress of overweight in children. Hence, our results may not give the full picture of parents’ knowledge about the presumed causes and progress of overweight in children.

Another important limitation of our study was its use of a non-validated questionnaire. However, a validation of the questionnaire was not possible within the frame of the study period. The questionnaire survey method affords us some insight into the beliefs and expectations of the parents. To gain a deeper insight into these aspects, the use of qualitative methods seems a useful supplement.

The GP distribution method was believed to yield superior response rates. However, it is likely that a recent encounter with the GP accentuated the role of general practice and we may consequently have overestimated the parents’ expectations towards their GPs.

The five-year PCHE was chosen as the focus of our investigations, as the participation rate is relatively high and it is the last PCHE in general practice before the municipal services take over at school start. The lower participation rate at a previous PCHE would most likely have caused further bias due to selection of parents; more engaged parents with more resources. It may, furthermore, be discussed whether the five-year PCHE is, indeed, the most appropriate time to focus on overweight in children. An evaluation of the PCHEs in Danish general practice showed that approximately one fourth of the parents found it important to address healthy lifestyle, i.e. diet and exercise, especially at the 12-month and five-year PCHE [2]. Moreover, our preceding qualitative interviews showed that the GPs encountered substantial barriers when raising the issue of overweight in children below the age of five.

### Clinical implications

Our findings have important implications for general practice. In the present study most parents expected the GPs to raise the issue of overweight in all children when relevant. This knowledge should help GPs gain confidence in their future care of overweight children. Our study should contribute to the breakdown of GPs’ barriers regarding parents not recognising the health risk of overweight in children.

Most parents agreed on diet and lack of exercise being causes of overweight. This emphasises the fact that losing weight is not only a matter of providing knowledge to the families. It rather seems that follow-up by the GP could be an important input as this is expected from most of the parents.

Parents of overweight children may have different beliefs of the causes and progress of overweight, which is important knowledge for the GP when communicating with parents about overweight in their child. Hence, GPs should be attentive to the particular beliefs of this group of parents and seek to explore their specific frames of reference.

#### Future research

A future interview study amongst PCHE non-participants would be instrumental in providing a more comprehensive picture of parents’ beliefs and expectations to GPs, making general practice able to meet the needs of this group of parents. Moreover, the beliefs and expectations of parents of overweight children should be further addressed in future research.

## Conclusion

According to parental beliefs and expectations, general practice should have an important role to play in the management of child overweight. Moreover, our findings suggest that GPs should be aware of the particular beliefs of parents of overweight children.

### Ethics

The study was approved by the Danish Data Protection Agency (j.nr. 2008-41-3239) and data are deposited according to standards in our research department. The study was recommended by the Multipractice Study Committee (MPU 10–2007). According to Danish legislation, no ethical approval is required when biological tests are not performed on the individuals under study.

All parents gave informed written consent to participation in the study.

## Abbreviations

GP: General practitioner; NOWC: Non-overweight children; OWC: Overweight children; BMI: Body mass index = kg/m^2^; WHO: World Health Organisation.

## Competing interest

The authors declare that they have no competing interests.

## Authors’ contributions

BC, JS and MKA designed the study. MKA analyzed the data. BC, JS and MKA interpreted the data and wrote the paper. All authors read and approved the final manuscript.

## Authors’ information

All authors are GPs. MKA authored the PhD thesis “Child overweight in general practice. GPs’ recognition and care. Parents’ beliefs and expectations”. JS and BC were supervisors on the thesis.

## Pre-publication history

The pre-publication history for this paper can be accessed here:

http://www.biomedcentral.com/1471-2296/14/152/prepub
